# Biomedical document triage using a hierarchical attention-based capsule network

**DOI:** 10.1186/s12859-020-03673-5

**Published:** 2020-09-17

**Authors:** Jian Wang, Mengying Li, Qishuai Diao, Hongfei Lin, Zhihao Yang, YiJia Zhang

**Affiliations:** Dalian University of Technology, The School of Computer Science and Technology, Dalian, 116024 China

**Keywords:** Biomedical document triage, Capsule network, Hierarchical attention mechanism, Biomedical literature

## Abstract

**Background:**

Biomedical document triage is the foundation of biomedical information extraction, which is important to precision medicine. Recently, some neural networks-based methods have been proposed to classify biomedical documents automatically. In the biomedical domain, documents are often very long and often contain very complicated sentences. However, the current methods still find it difficult to capture important features across sentences.

**Results:**

In this paper, we propose a hierarchical attention-based capsule model for biomedical document triage. The proposed model effectively employs hierarchical attention mechanism and capsule networks to capture valuable features across sentences and construct a final latent feature representation for a document. We evaluated our model on three public corpora.

**Conclusions:**

Experimental results showed that both hierarchical attention mechanism and capsule networks are helpful in biomedical document triage task. Our method proved itself highly competitive or superior compared with other state-of-the-art methods.

## Background

Biomedical natural language processing (BioNLP) has an important role in the framework for implementing precision medicine [1–3]. Biomedical document triage is an important task in BioNLP, and is the first step in the literature curation workflow [4, 5]. Biomedical document triage helps curators and researchers focus on the biomedical literature that contains information relevant to their tasks [6, 7].

In the past decade, biomedical document triage has been an important shared task in the BioCreative challenge community. For example, BioCreative II (IAS) [8] and III (ACT) [9] focused on the classification of whether a given article contains protein interaction information. BioCreative VI (PM) [10] focused on identifying relevant PubMed citations describing genetic mutations affecting protein-protein interactions. Similarly, various methods have been proposed for the task of biomedical document triage [11]. The majority of these tasks can be divided into either machine learning-based methods or neural network-based methods.

As for machine learning-based methods for biomedical document triage, most depend on effective feature engineering including lexical and syntactic information. For example, Si L et al. [12] utilized logistic regression and support-vector machine algorithms to generate ranked lists of documents. Almeida H et al. [13] experimented with dataset sampling factors and a set of features, as well as three different machine learning algorithms including naive Bayes, support-vector machine and logistic model trees. Generally, machine learning-based methods are skill-dependent and labor-intensive, requiring lots of effort to design particular features.

Recently, neural network-based methods [14] have been successfully applied to biomedical documents. Kim et al. [15] reported on a series of experiments using convolutional neural networks (CNN) trained on top of pretrained word vectors for sentence-level classification tasks. Lai et al. [16] introduced a recurrent convolutional neural network for text classification, which combines CNN with a recurrent neural network (RNN). Some of the above-mentioned methods have been successfully applied to biomedical document triage [17–19]. Attention mechanisms, which can capture the relatively important parts of the input text, have been successfully applied in BioNLP [20, 21].In 2016, Yang et al. proposed a two-layer attention network for text classification [22]. That network would obtain the characteristics of both words and sentences within a document. In 2017, Hinton proposed the CapsNet network architecture [23], which was based on the traditional CNN but it modified some layers.

However, while these methods can automatically extract features to save time and energy in the documents triage task, they have limitations in dealing with long biomedical documents. In the BioCreative VI Precision Medicine Track of the triage task, even the top team received an F-score of less than 70 percent [10]. Those models mentioned above cannot effectively learn the latent feature representation from long biomedical texts. Considering that the word-level attention layer can capture the internal association in the sentence. The obtained vector can reflect the global feature with the information about all the words of the entire sentence. The sentence-level attention layer can capture the association feature between sentences in an article. Recent studies [23–25] indicates that the capsule network retains the advantages of CNN and improve its shortcomings. It can capture more information on spatial patterns aggregated at lower levels that contribute to representing higher level concepts. It forms a more effective recursive process to articulate what to be modeled when there is less training dataset. In general, document text is much longer than sentence text. In particular, some biomedical document texts contain several very complicated sentences including medical terms. In our study, we make full use of the complementarity of hierarchical attention and capsule network to construct our model, in which the attention mechanism can reduce the problem of dependence in-formation loss in the long biomedical document text and the capsule network can capture more feature information at lower levels even there are complicated sentences in biomedical document. These recent advances in neural networks may improve the performance of biomedical document triage. Both attention mechanisms and capsule networks may be helpful in biomedical document triage.

In this paper, we propose a hierarchical attention-based capsule network model, which can effectively capture the important features across sentences and learn the comprehensive latent feature representation for the whole document text. Firstly, our model employed the dynamic route algorithm to accurately identify more features in the biomedical text and improve precision instead of using a max-pooling method where important information may be lost to filter features. Additionally, a hierarchical attention mechanism was used to capture valuable features at both the sentence-level and word-level to better deal with the long text of a document. Our method was evaluated on three public corpora including BioCreative VI Precision Medicine (PM) corpus, BioCreative II (IAS) corpus and BioCreative III (ACT) corpus. Experimental results suggest that the proposed model achieved state-of-the-art performance on all three corpora.

The rest of paper is organized as follows. In “Methods” we give a brief introduction of the biomedical document triage task and describe our proposed model in detail. Then, we present and discuss the experimental results on the three corpora in “Results and discussion”. Finally, our conclusion and future plans are presented in “Conclusion” sections.

## Methods

### Biomedical document triage

Biomedical document triage is generally approached as the task of classifying whether a specific article contains information relevant to what is needed. In this paper, we choose three public corpora: BioCreative VI Precision Medicine (PM) corpus, BioCreative II (IAS) corpus and BioCreative III (ACT). All three corpora have been examined by biological curators and domain experts.

**Fig. 1:**
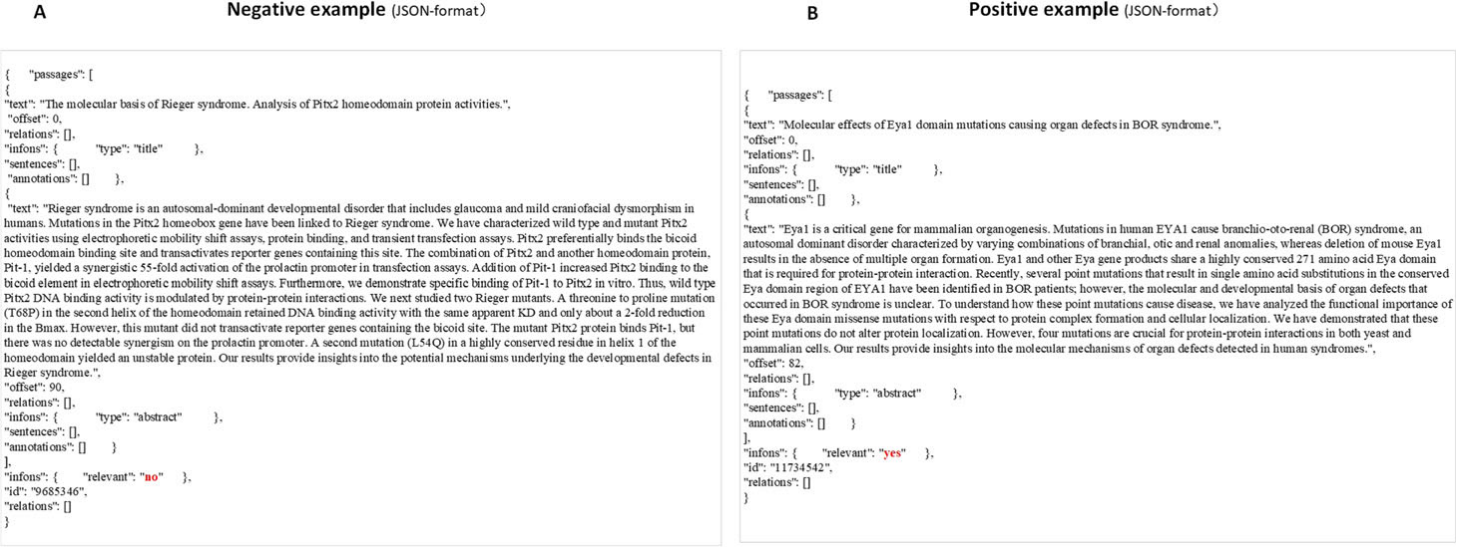
Negative examples and positive examples of JON-format from PM datset. **a** negative examples of JON-format from PM datset. **b** positive examples of JON-format from PM dataset

The PM corpus is provided by the BioCreative VI Precision Medicine Track task [10]. The PM corpus contains training and test sets that are stored in the ’JSON’ format, Fig. 1 gives a negative example and a positive example of the PM corpus. The corpus file includes two types of passages. One is the title text and the other is the abstract text, the latter of which is marked by the label of ‘infons’. Negative examples are annotated with a ’no’ under the ’relevant’ label, this annotation helps identify text that is relevant to our area of interest, genetic mutations affecting protein-protein interactions (PPIm). All selected articles were manually annotated by the official organization to answer these questions: Does this article describe experimentally verified protein-protein interactions? Are the database curated PPI pairs for this article mentioned in the abstract? Does this article describe a disease known mutation or a mutational analysis experiment? Is the PPI affected by the mutation? Then, based on the above annotations, articles are carefully categorized as 1)Positives, for articles specifically describing PPI influenced by genetic mutations, 2)Negatives, for articles describing both PPIs and genetic variation analysis with no inference of relation between them or containing PPI but no mutations or containing mutations but no PPI or mentioning neither [10]. In the training set of the PM corpus, there were 1729 relevant articles that describe the protein-protein interactions affected by genetic mutations and 2353 articles that were not relevant. Lastly, the PubMed Unique Identifier (PMID) (e.g. 9685346) of the passages is given under the ’id’ label.

**Fig. 2:**
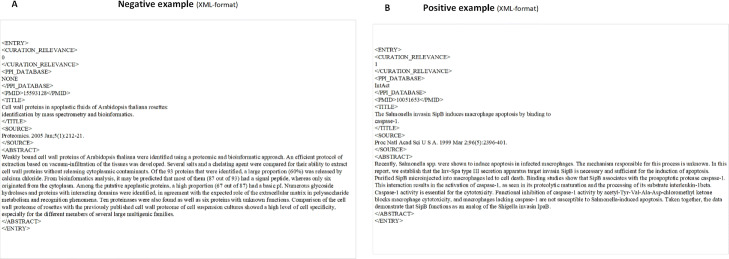
Negative examples and positive examples of XML-like format from IAS dataset. **a** negative examples of XML-like format from IAS dataset. **b** Positive examples of XML-like format from IAS dataset

The IAS corpus and ACT corpus are a little different from the PM corpus. They are provided in the XML-format. The IAS corpus file includes training and test sets. The IAS training set corpus contained 3536 positive examples that were relevant to protein interactions and 1959 negative examples that were not relevant to protein interactions. The IAS test set contained 338 positive examples and 339 negative examples. The ACT corpus file includes training, development and test sets. There were 1140 positive examples and 1140 negative examples in the ACT training set, 682 positive examples and 3318 negative examples in the ACT development set, and 910 positive examples and 5090 negative examples in the ACT test set. Figure 2 gives an illustration of positive and negative examples of XML-like corpus format. The label of positive and negative is under the ’CURATIONRELEVANCE’ label. The corpus in the XML-format also gives the PMID, title text, abstract text and so on under the relevant labels.

### The model architecture

**Fig. 3:**
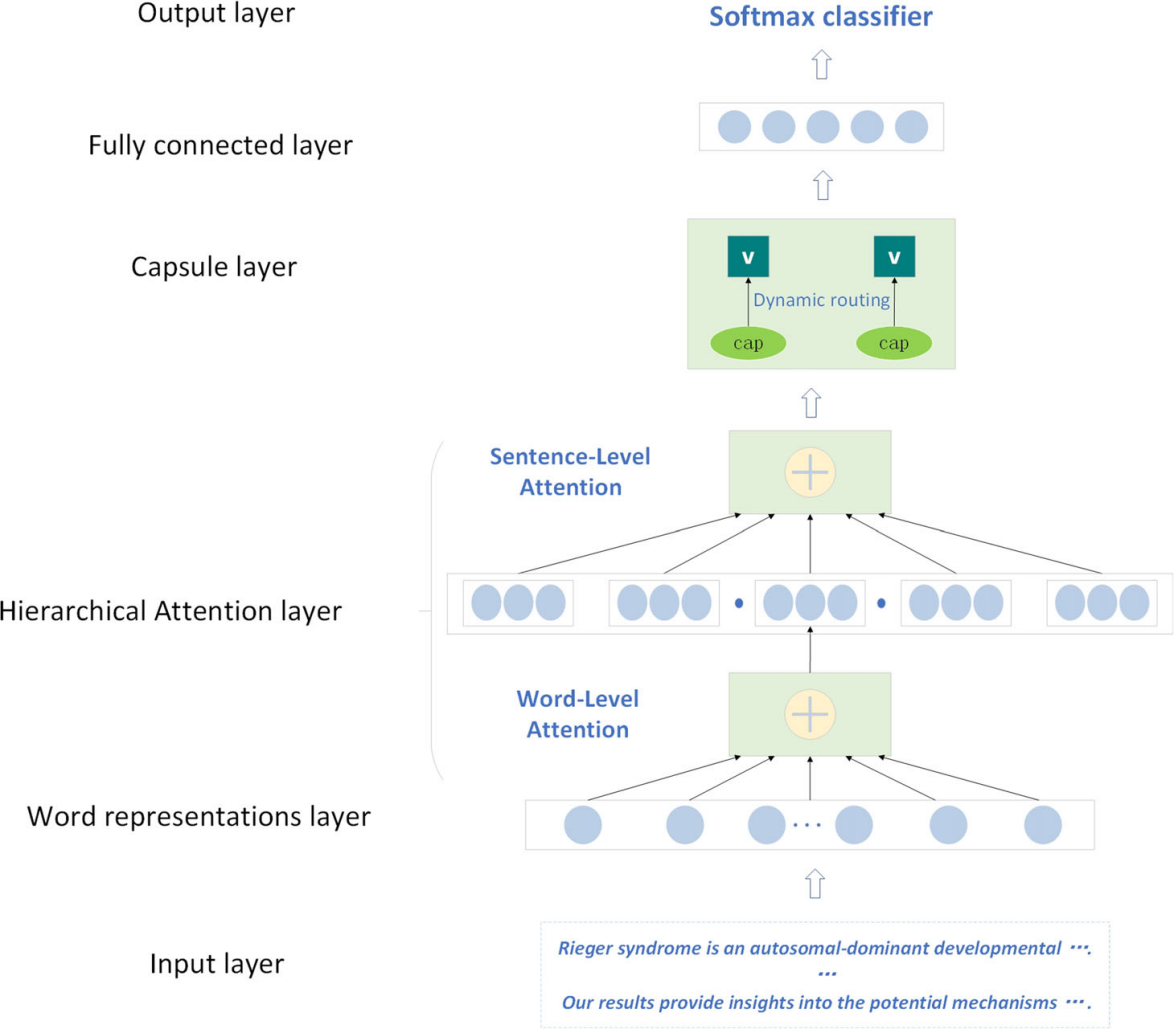
The schematic overview of our model

An architecture schematic overview of our model is shown in Fig. 3. In general, our model consists of three main parts: the hierarchical attention layer, the convolution neural network (CNN) and the capsule network layer. The inputs of our model are text sequences. The word embedding generates the distributed representation vector including semantic information for each word. The hierarchical attention layer applies a sentence-level and a word-level multi-attention mechanism to capture the relatively important features based on the whole word representations from the two levels in the long text. After the hierarchical attention mechanism layer, a convolution layer is used to learn some local features from the long text. Importantly, in order to prevent losing significant features to the max-pooling operation of the CNN, we use the dynamic routing algorithm in the capsule layer and convert scalar feature output to vector feature output to learn more features. At last, we employ a fully connected layer and the *Softmax* function to implement document triage.

### Word representations

The distributed representation, also known as word embedding [26, 27], is based on the hypothesis that semantically similar words have similar semantics. In the field of BioNLP word embedding is widely used, it effectively captures the semantic information underlying each word. For this paper, we used the pre-trained word embedding downloaded from https://github.com/cambridgeltl/BioNLP-2016, which was trained on the PubMed Central Open Access subset (PMC) corpus.

### Hierarchical attention mechanism

Attention mechanisms have become an important part of some compelling sequence models and transduction models for various tasks, allowing modeling of dependencies without regard for their distance in the input or output sequences [22, 28, 29]. In our model, we combine word-level and sentence-level attention mechanisms to capture important features across sentences.

#### Multi-head attention mechanism

**Fig. 4:**
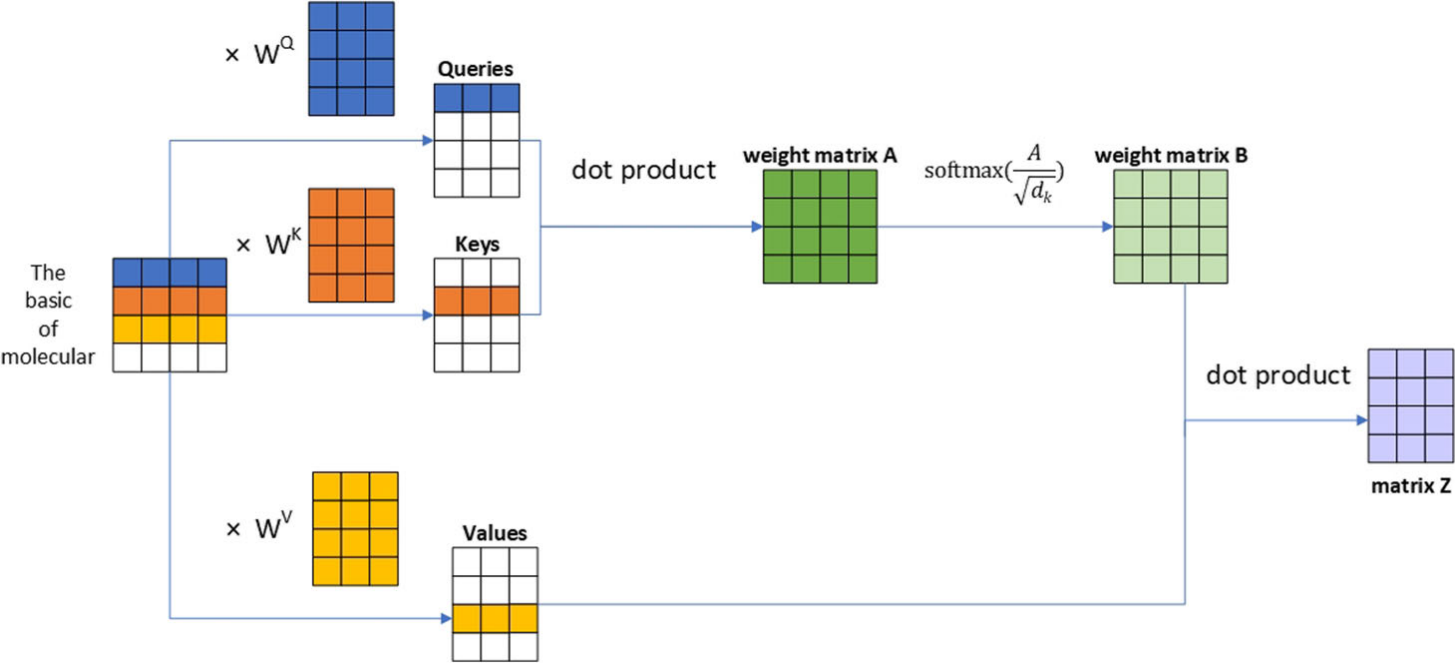
The self-attention mechanism calculation process. We can get three vectors that are a query vector, a key vector and a value vector for each word by multiplying the embedding word vector by the three matrices trained during the training. The size of these new vectors is 3, while the dimensions of the embedding word and encoder are 4. We evaluate the dependence between the words with the dot product operation

The principle on which the multi-head attention mechanism functions is that applying the attention many times may learn more features than a single application. For attention mechanisms, the self-attention mechanism is a special case where the input and output are the same sequences in the Encoder-Decoder framework. The physical meaning in machine translation is a word alignment mechanism between the target word and the source word in the general attention mechanism, while the self-attention mechanism learns the internal connection or grammatical structure of the sentence. As an example, we consider the sentence “The animal didn’t cross the street because it was too tired.” What does “it” refer to, “street” or “animal?” This is a simple problem for humans, but it is not simple for an algorithm. When the model processes the word “it,” the self-attention mechanism associates “it” with “animal.” When the model processes each word, that is, when processing each position of the input sequence, the self-attention mechanism allows it to look at other locations in the input sequence to find ways to better encode each word. Figure 4 illustrates the calculation process of the self-attention mechanism.

An attention function is created by mapping a query and a set of key-value pairs to an output, where the key, values, query and output are all vectors. A weighted sum of the values is the output, where the weight assigned to each value is computed by a compatibility function of the query with the corresponding key. Our input was made of queries and keys of the dimension *d*_*k*_, and values of the dimension *w*. The dot products of the query with all keys were computed and each was divided by $\sqrt {d_{k}}$. Finally, a *Softmax* function was be used to obtain the weights of the values, which is shown in the following formula.

$$ Attention(Q,K,V)=softmax\left(\left(QK^{T}\right)/\sqrt{d_{k}}\right)V $$

where *Q* is a matrix in which a set of queries is packed together. Similarly, *K* and *V* are the matrices in which the keys and values, respectively, are packed together.

It is more effective to linearly project the queries, keys, and values many times with different dimensions for each than to perform a single attention function with the dimensional keys, values, and queries, which is the main idea of the multi-head attention mechanism. The model is allowed to jointly attend to information from different representation subspaces at different positions in the multi-attention mechanism, which is shown as follows:

$$head_{i}=Attention\left(QW_{i}^{Q},KW_{i}^{K},VW_{i}^{v}\right) $$

$$ MultiHead(Q,K,V)=Concat\left(head_{1},\ldots,head_{h} \right) W^{o} $$

where the $W_{i}^{Q}, W_{i}^{K}$ and $W_{i}^{v}$ are the parameter matrices, whose dimensions are *d*_*m*_*odel*×*d*_*k*_,*d*_*m*_*odel*×*d*_*k*_, and *d*_*m*_*odel*×*d*_*v*_, respectively. The dimension of *W*^*o*^ is *hd*_*v*_×*d*_*m*_*odel*, where *h* is the number of times a single attention calculation is performed. The matrices above all are initialized randomly. After training, each set of input vectors is projected into a different representation subspace.

The multi-head attention mechanism improves the performance of the attention layer by extending the model’s ability to focus on different locations and provide multiple “representative subspaces.” Our goal is that the attention model will learns different dependency information in different heads from the long biomedical texts so that we can further improve on the performance of the biomedical text classification task.

#### Hierarchical attention mechanism

Hierarchical attention is aimed to capture two basic types of features from a biomedical document structure, one being word-level features and the other being sentence-level features.

Since the time complexity of the attention mechanism is *O*(*n*2), the amount of computation increases significantly when the input of the model is a longer text. On the other hand, there is little connection between words found in different sentences or long sentences. There are the word-to-sentence and sentence-to-document features in each text. Correspondingly, the representation of the sentence can be constructed first by the word, followed by a representation of the text that can be constructed by the sentence. Because different words and sentences have different information, not only can the information between the words be obtained, but the information between the sentences can be obtained with the two-levels of features. Hierarchical attention mechanisms can give words and sentences different weights to accommodate that fact the same words and sentences can have different roles in different texts. The problem that dependence information is lost when the input text is too long, which often occurs in the text classification task, can be solved by the hierarchical attention mechanism and we can get more features from words and sentences in the document in this way.

**Fig. 5:**
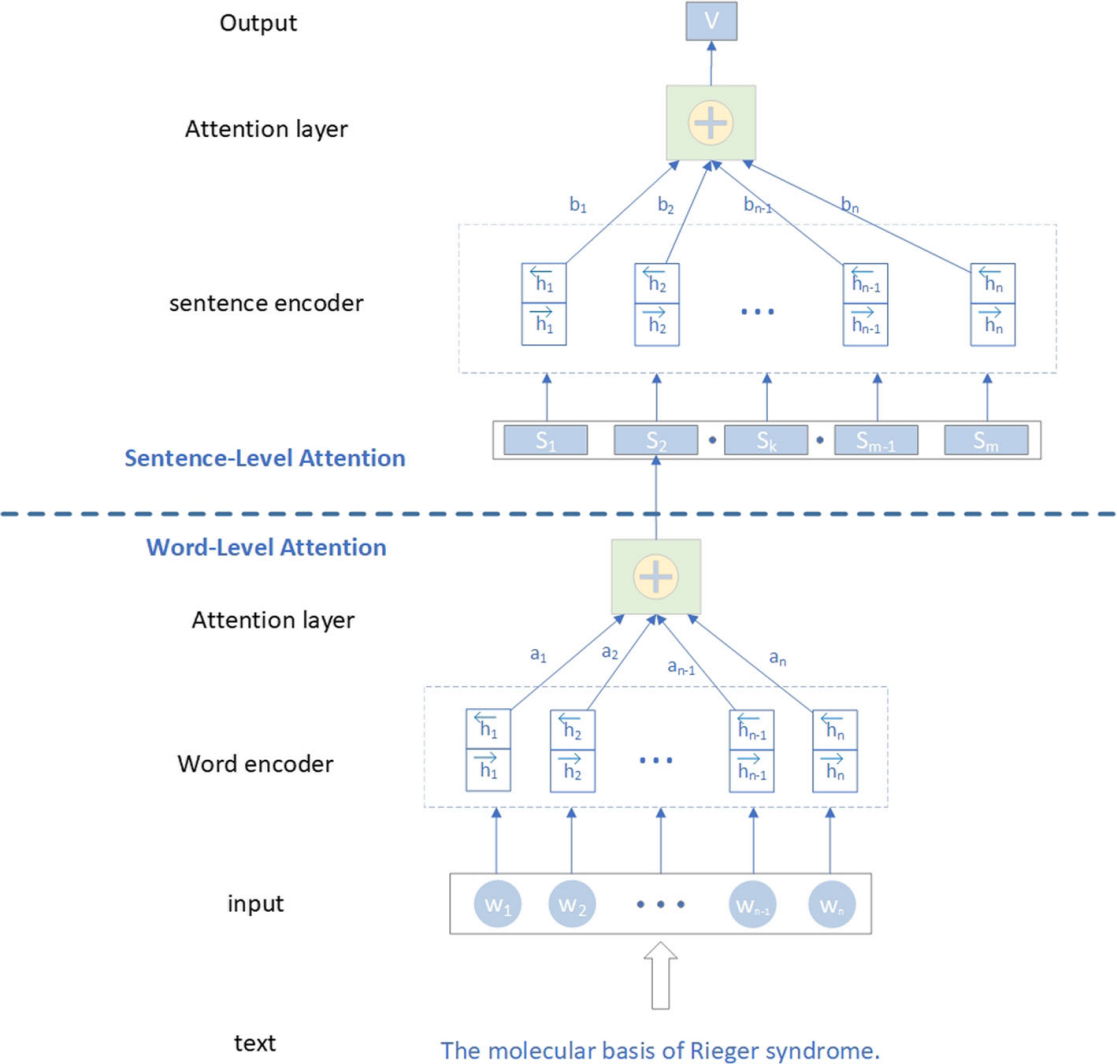
The hierarchical attention mechanism architecture. We connect the word-level and sentence-level features by attention mechanism to get a feature vector with more information and the Softmax function is used to normalize the result

There are many sentences in each text and many words in each sentence, complicating the biomedical document triage task, this makes the training speed slow when using the self-attention mechanism directly. In our work, we mainly use hierarchical attention mechanisms based on self-attention. First, the self-attention mechanism at the word level is used to find the dependencies between words in the sentence, and then it is used at the sentence level to find the dependencies between the sentences in the document, which not only speeds up the training, but also establishes the characteristics of the word and sentence levels in the text. Our experimental result shows its effectiveness in improving the performance of the document triage task. We use the first part of the hierarchical attention model to process the words of each clause with the purpose of transforming the sentences into vectors. The second part deals with the sentence vector of the document to generate a new sentence vector using the self-attention calculations and perform subsequent convolution operations. Figure 5 gives the hierarchical mechanism architecture. We can see that the input text is split into words (*w*_1_,*w*_2_,…,*w*_*n*_) that will be encoded by the recurrent neural network. Each word will get an attention weight (*a*_1_,*a*_2_,…,*a*_*n*_) about the input text sequence. Then the output of the word-level attention will be used as the feature of the sentence-level attention for the sentence encoder. The sentence-level attention input is the document which is split into sentences (*s*_1_,*s*_2_,…,*s*_*n*_), and we will calculate the attention weights (*b*_1_,*b*_2_,…,*b*_*n*_) for the sentences. At last, we will get a feature vector with more information and the *Softmax* function will be used to normalize the result.

### Convolution neural network (CNN)

After the hierarchical attention layer, we use a slight variant of the traditional convolutional layer as the baseline to further capture local features for optimization of our model. Generally, each convolutional layer of the traditional CNN is serially connected, that is, the output of the upper layer is used as the input of the next layer, but in our experiment, each convolutional layer of the CNN is connected in parallel, and the output of each convolutional layer is spliced together as the output of the CNN. In this layer, let *x*_*i*_ be the k-dimensional word vector corresponding to the *i*−*th* word in the sentence. A sentence of length n is represented as follows. If the length is not n, we will pad it where necessary.

$$x_{(1:n)} = {x_{1},x_{2},\ldots,x_{n}} $$

*x*_(1:*n*)_ denotes the concatenation operator of *x*_1_,*x*_2_,…,*x*_*n*_. The convolution operation can be seen as filtering features, which obtains local optimal features through the kernel function. Then these features are combined together to form new features. In this way, each layer is filtered out and the more significant features are passed to the higher layers to calculate as follows:

$$S_{((t))}= ReLU\left(Wx_{(t:t+w-1)}+b\right) $$

where *W* is a transformation matrix, also known as the convolution kernel function. The input sequence is [*x*_*t*_,*x*_(*t*+1)_,…*x*_(*t*+*w*−2)_,*x*_(*t*+*w*−1)_], where the lowercase *w* is the input window size. *ReLU* is an activation function that is a non-linear unit function and *b* is the bias vector. The filter is applied to each possible window of words in the sentence *x*_(*t*:*t*+*w*−1)_ to produce the feature map S, which is the convolutional layer result.

### Capsule network

A CNN model can effectively capture local features, but cannot capture global features. In brief, a CNN generates different features through multiple convolution kernels, and the features are accumulated layer by layer, but in this process, the network loses important information: i.e. the spatial relationship between the features. To address this disadvantage of the CNN model, Hinton et al. proposed capsule networks [23]. An important concept of CNN is the pooling strategy, which downsamples the input vectors. In the text classification area, each convolution kernel can be used to detect the relevant meanings of consecutive words to generate text features. If a similar text feature reappears, the output value of this convolution kernel becomes larger, which is well preserved by the pooling strategy. The pooling can handle the translation change. When a feature moves, as long as it does not exceed the size of the pooled window, it will not be lost and will be detected, which can make the network position-invariant. However, the disadvantage of this method is that the pooling operation, such as max-pooling, retains only the most important features while losing a lot of information. The ideal pooling not only reduces the data dimension, but also retains various features and information so that each feature does not change through the pooling layer. Based on this idea, the capsule network replaces the scalar-output feature detectors of CNN with vector-output capsules and max-pooling with routing-by-agreement, it still likes to replicate learned knowledge across space. Unlike max-pooling, information about the precise position of the entity within the region is not thrown away in the capsule network.

The capsule network is well trained by a powerful dynamic routing mechanism that ensures the capsule’s output reaches the appropriate parent node. The basic idea of the dynamic routing mechanism is to design a nonlinear mapping strategy, whose task it is to let the output reach the appropriate parent capsule. More specifically, the capsule output is sent to all of the parent capsules in the next layer, and then, for each possible parent capsule, the sub-capsules calculate their outputs by multiplying by the weight matrix. If this result is a large scalar product of the parent capsules output, then the connection between the sub-capsule and the parent capsule should be close, which is achieved by increasing the coupling coefficient of the sub-capsule to the parent capsule and reducing the coupling coefficient with other parent capsules. In theory, this dynamic routing protocol is more efficient than the routing method implemented by max-pooling. From a mathematical point of view, a non-linear “squashing” function is used to ensure that short vectors get shrunk to a near zero length, which is shown as follows.

$$v_{j}=\left(||s_{j} ||^{2}\right)/\left(1+||s_{j} ||^{2}\right) s_{j}/\left(||s_{j} ||\right) $$

where *v*_*j*_ is the vector output of capsule *j* and *s*_*j*_ is its total input. For all but the first layer of the capsules, the total input to a capsule *s*_*j*_ is a weighted sum over all “prediction vectors” $\widehat {u}_{\left (j|i\right)}$ from the capsules in the layer below and is produced by multiplying the output *u*_*i*_ of a capsule in the layer by the weight matrix *W*_(*ij*)_, which is shown as follows.

$$s_{j}=\Sigma_{i} c_{\left(ij\right)} \widehat{u}_{\left(j|i\right)} $$

$$\widehat{u}_{(j|i)}=W_{\left(i j\right)} u_{i} $$

where the *c*_*i*_*j* are coupling coefficients that are determined by the iterative dynamic routing process. The calculation method of *c*_*i*_*j* is shown as follows.

$$c_{\left(ij\right)}=exp\left(z_{\left(ij\right)}\right)/\Sigma_{i} exp\left(z_{(ik)}\right) $$

The coupling coefficients between capsule *i* and all the capsules in the layer above are determined by a routing *Softmax* whose initial logits *z*_*i*_*j* are the log prior probabilities that capsule *i* should be coupled to capsule *j*. The coupling coefficients sum to 1.

**Fig. 6:**
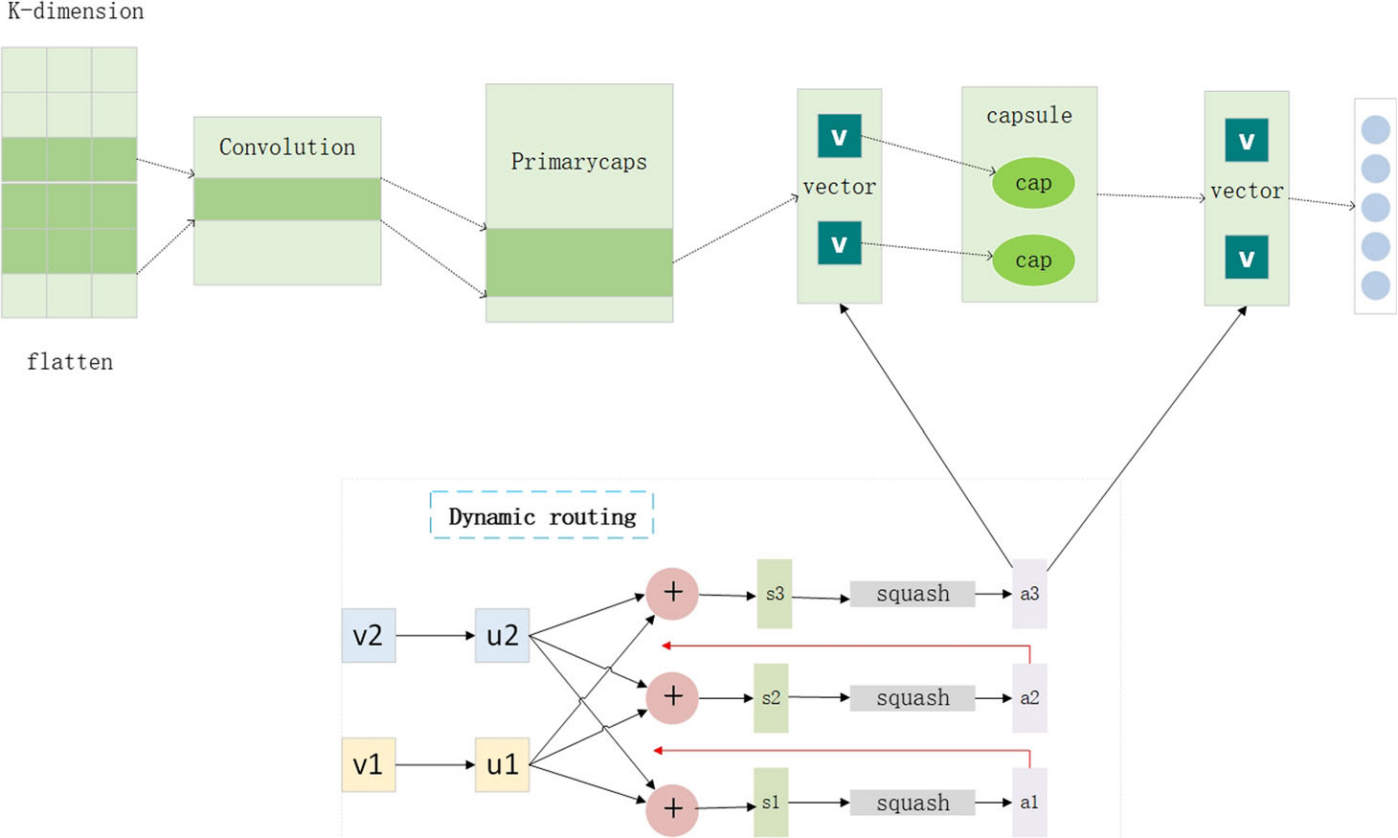
The capsule network model architecture

Our capsule network architecture is shown in Fig. 6. It can be seen that we first use the convolutional layer to extract some primary features and next use a primary capsule layer to capture more features and convert the scalar output to vector output to be the input of the next capsule layer which can get accuracy features. The useful information in the biomedical long texts can be preserved by the dynamic routing algorithm that is used in our two capsule layers. In the capsule network, the output is a vector instead of a scalar, this means that the output of the primary capsule layer will give a very clean and accurate signal to the appropriate subsequent capsule for the exact transmission of information. The capsule vector dimension is set to 32 in our experiments and the number of the dynamic routing iterations is set to 3 which can be seen from Fig. 6. In the dynamic routing algorithm, the vectors (*v*_1_,*v*_2_) are the input which is transformed by the affine transformation to get the outputs *u*_1_,*u*_2_. The *s*_1_,*s*_2_,*s*_3_ will be calculated as the sum of *u*_1_,*u*_2_. Then, *s*_1_,*s*_2_,*s*_3_ will be used in formula to calculate the vector outputs (*a*_1_,*a*_2_,*a*_3_). The results of *a*_1_,*a*_2_ will be fed back to optimize the last result *a*_3_.

### Classification and training

We used the ^′^*Softmax*^′^ function on the output layer to implement the detection and classification of the biomedical documents about PPI and PPIm in this paper. In the experiments, the programming language was Python, and the version was 3.5. The python libraries we used included numpy, scikit-learn, gensim, etc. Our model was implemented by Keras with the TensorFlow 1.13.1 backend. We employed the dropout layer mechanism before the word representation layer and output layer to prevent the overfitting of the neural network model. The hyperparameters used in our experiments are listed as follows. The dimension of our models in the word embedding was initialized to 200 to adapt to the time and space computational complexity. The number of hidden layer neurons was set to 100. The batch size was set to 128 and the number of epochs was set to 50 during the training. The size of each convolution kernel was 3, 4, and 5, which was set to 128 feature maps, and the dropout probability was set 0.5 and 0.8. The convolutional layer activation function was a ReLU function. In the hierarchical attention layer, the heads number of the first attention mechanism layer was set to 8, the vector dimension was set to 32, the second layer of attention mechanism had a headcount of 8, and a vector dimension of 32. In the capsule neural network, the dimension was set to 32, and the dynamic route iteration number was 3. The learning rate was set to 0.01. Then, all parameters of the models were optimized by using Adam [30] to minimize the categorical cross-entropy loss. The computing environment is Ubuntu 16.04.5 LTS and the hardware environment is GeForce GTX Titan Xp. Our source code is available at https://github.com/dqshuai/text_classification-of-biomedicine.

## Results and discussion

### Datasets and evaluation metrics

In our experiment, we used the corpus from the BioCreative II Interaction Article Sub-task, BioCreative III Article Classification Tasks and the BioCreative VI Precision Medicine Track to mine for protein interactions and mutations for a precision medicine task. The statistics of all corpus are presented in Table 1.

**Table 1:** Dataset statistics

Corpus	Positive	Negative	Total
PM Training set	1729	2353	4028
PM Test set	704	723	1427
IAS Training set	3536	1959	5495
IAS Test set	338	339	667
ACT Training set	1140	1140	2280
ACT Development set	682	3318	4000
ACT Test set	910	5090	6000

In the PM corpus experiments, 10% of the PM training sets were randomly selected as the development set to tune the models’ hyperparameters and the remaining data were used to train our model. We evaluated the models on the open test set provided by the organizers of the BioCreative VI PM document triage task. We proved the effectiveness of our model by testing it on the other corpus, the test sets of IAS and ACT.

The Precision (P), Recall (R) and F-score (F1) were used to measure our model’s performance on the biomedical document triage task of PM corpus and IAS corpus, which were calculated by the official evaluation scripts. P, R, and F were calculated as follows:

$$P=TP/((TP+FP)) $$

$$R=TP/((TP+FN)) $$

$$F1=2PR/((P+R)) $$

The F1 value is a comprehensive evaluation of the precision and the recall, which is an evaluation the overall performance. TP compares the actual positive examples to the positive examples that the model identified correctly, known as true positive examples, FP compares the actual negative examples to the positive examples that the model identifies incorrectly, known as false positive examples, and FN compares the actual positive examples to the negative examples, known as false negative examples. We can use the confusion matrix to express those clearly.

### Performance of our method

#### Baseline methods

Before inputting text into the model, some preprocessing of the original corpus was conducted. First, we extracted the texts and lables from the original corpora. Secondly, the text data was removed some invalid data points, such as those with empty text. Moreover, we use regular matching to keep only the letters and numbers. Then we began to process the sentence-level and word-level corpus to prepare for input into the model. For the input data of the attention mechanism, the extracted text needed to be split into sentences, then the sentences were split into words, and some noisy sentences were selectively removed. In order for the length of each text to be consistent when the model was trained, text padding was performed with <*PAD*/>.

We compared our biomedical document triage method with some baseline methods, including CNN, capsule network, and self-attention, using the word em-bedding trained by Word2Vec tool as the model input.

CNN: This is a traditional method for text classification. In our work, this network consisted of the word representing layer, convolution layer, max-pooling layer, dropout layer, dense layer, and *Softmax* layer, successively. We used two convolution layers for the 128 feature maps, which were learned for each of two different filters size 3,8 and the step size is set to 2 in the max-pooling layer. The parameters of the dropout layer were set at 0.5 and 0.8.

Capsule network: This method was first used to recognize highly overlapping digits. In our work, we use the capsule network based on a CNN to process the text, which is for the BioNLP field. We used the neuron vectors to replace the neuron scalar nodes in the traditional deep neural network and used the dynamic routing protocol to replace the max-pooling layer in the CNN to train the new neural network. The capsule vector dimension was set to 32, and the dynamic route iteration was set to 3. Therefore, the capsule network was made up of the word representing layer, convolution layer, capsule layer, dropout layer, dense layer, and *Softmax* layer, successively.

Self-attention: Self-attention was first used in machine translation tasks to surpass and replace the recurrent neural network. In our baseline work, we combine the CNN with the self-attention to classify biomedical documents. We set the number of multi-head attention heads at 8 and the vector dimension of the attention mechanism at 64. This neural network was made up of a word embedding representation, self-attention layer, convolution layer, dropout layer, dense layer, and *Softmax* layer, successively.

The results of the baseline methods are shown in Table 2. From Table 2, we can make the followings observations. Firstly, CNN only used the language pretraining model trained by Word2Vec tool to get information from the text, which achieves an F-score of 0.664. Secondly, the capsule network effectively improved the F-score from 0.664 to 0.686. The results indicate that the dynamic routing algorithm of capsule networks was able to capture more features from the text information. Thirdly, integrating self-attention can significantly improve the performance of the CNN model (by an average improvement of 4.3% in F-score). The experimental results suggest that both capsule networks and attention mechanism are helpful in biomedical document triage task.

**Table 2:** The results of baseline methods

Methods	P	R	F1
CNN	0.581	0.774	0.664
Capsule network	0.629	0.755	0.686
Self-Attention	0.584	0.895	0.707

#### Effects of hierarchical attention mechanism

In Table 3, we evaluated the effect of a hierarchical attention mechanism on the PM corpus. From Table 3, we can see that the performance of the hierarchical attention mechanisms based on CNN was significantly higher than the CNN alone (an average improvement of 5.1% in F-score) and the self-attention based on CNN (an average improvement of 0.8% in F-score). The experimental results show that the hierarchical attention mechanisms greatly improved the precision value and captured more dependency features between words and sentences than the self-attention mechanism which only mines word-level information. The attention mechanism can reduce the problem of dependence information loss in the long biomedical document text.

**Table 3:** The results of hierarchical attention

Methods	P	R	F1
CNN	0.581	0.774	0.664
CNN+self-attention	0.584	0.895	0.707
CNN+hierarchical attention	0.623	0.840	0.715

#### Effects of capsule network

In Table 4, we evaluated the effect of capsule networks on the PM corpus. From Table 4, we can see that the single capsule network performed better than the single CNN (an average improvement of 2.2% in F-score). The capsule network based on hierarchical attention achieved better performance than and the CNN based on the hierarchical attention (an average improvement of 0.8% in F-score). The proposed hierarchical attention-based capsule network model achieved the best performance in all methods when we use the capsule network based on hierarchical attention, which had an average improvement of 5.9% in F-score compared to the baseline of CNN. The precision and recall were both improved by this method. Hierarchical attention can cover the shortage of the self-attention in precision while the capsule network can further improve the hierarchical attention in recall. The capsule network can capture more feature information at lower levels even there are complicated sentences in biomedical document.

**Table 4:** The results of capsule network

Methods	P	R	F1
CNN	0.581	0.774	0.664
Capsule network	0.629	0.755	0.686
CNN+hierarchical attention	0.623	0.840	0.715
CapsNet+hierarchical attention	0.624	0.895	0.723

### Performance comparison on PM corpus

In Table 5, we compared our method with other state-of-the-art methods on the PM corpus. It should be noted that the PrecMed Baseline [10] is the baseline method in the BioCreative VI PM document triage task and the PrecMed-best [10] is the method that got the highest F-score in the BioCreative VI PM document triage task challenge. “Team 418” got the highest precision and “Team 421” got the highest recall in the BioCreative VI PM document triage task challenge team competition. To the best of our knowledge, the ensemble model has had the best performance to date. Many deep learning models were used to improve the performance including five individual neural network models including LSTM (long-short term memory), CNN, LSTM-CNN (combine the LSTM and the CNN), recurrent CNN, and hierarchical LSTM. At last, they got an F-score of 0.710 by combining five models’ results with three different alternatives.

**Table 5:** Performance compared with other methods on PM corpus

Methods	P	R	F1
PrecMed Baseline [10]	0.610	0.636	0.622
Team 418	0.629	0.766	0.691
Team 421	0.570	0.874	0.690
PrecMed-best [10]	0.603	0.821	0.695
Ensemble model	0.629	0.815	0.710
CapsNet+hierarchical attention	0.624	0.895	0.723

Compared with other methods, our method has achieved the highest F-score (0.723) on the PM corpus. From Table 5, we can see our model’s F-score is 2.8 percentage points higher than the best performance in the BioCreative VI PM challenge. In particular, our method achieved an improvement of 1.3% over the ensemble model (0.710 F-score). What’s more, our model is relatively simple to implement unlike the ensemble model where high effort is required to construct the neural network models, which need much more time and energy to combine and integrate. To our knowledge, it is the first time to explore the complementarity of hierarchical attention and capsule network to classify long biomedical texts.

### Performance of our model on ACT corpus and IAS corpus

To demonstrate the generalization of our methods, we added some comparison experiments on the other corpus. One is the ACT corpus from the BioCreative III Article Classification Task and the other is the IAS corpus from the BioCreative II Interaction Article Sub-task. The corpus statistics are shown in Table 1.

We evaluated our hierarchical attention-based capsule model on the ACT corpus. The results of our method compared with the top teams in the BioCreative III ACT challenge are shown in Table 6. The participating teams were provided with a training set of 2,280 abstracts and a development set of 4,000 abstracts, while the evaluation was carried out on a test set of 6,000 abstracts through comparison to manual labels generated by domain experts. They measured the performance of the ten participating teams in this task over a total of 52 runs. Table 6 gives the best result of each participating team in their many runs. The experimental results show that our method achieves an F-score of 0.618 on the ACT corpus, which outperforms the top teams in BioCreative III ACT challenge.

**Table 6:** Performance compared with other methods on ACT corpus

Methods	P	R	F1
Team 65	-	-	0.598
Team 70	-	-	0.549
Team 73	-	-	0.614
Team 81	-	-	0.311
Team 88	-	-	0.344
Team 89	-	-	0.608
Team 90	-	-	0.596
Team 92	-	-	0.572
Team 100	-	-	0.594
Team 104	-	-	0.539
Our method	0.570	0.676	0.618

We also evaluated our model on the IAS corpus, shown in Table 7. Table 7 gives the best result achieved by each participating team. From Table 7, it can be seen that most of the teams achieved a high recall, but a modest precision. Our method also achieved the best F-score on the IAS corpus. All in all, our method is superior to the other state-of-the-art methods when applied to these three public corpora.

**Table 7:** Performance compared with other methods on IAS corpus

Methods	P	R	F1
Team 4	0.712	0.792	0.750
Team 6	0.708	0.860	0.777
Team 7	0.684	0.858	0.761
Team 11	0.676	0.781	0.725
Team 14	0.746	0.470	0.757
Team 19	0.645	0.565	0.602
Team 27	0.607	0.852	0.709
Team 28	0.750	0.810	0.779
Team 30	0.686	0.789	0.643
Team 31	0.667	0.594	0.629
Team 37	0.575	0.946	0.715
Team 41	0.619	0.890	0.730
Team 44	0.688	0.868	0.764
Team 48	0.588	0.863	0.700
Team 49	0.526	0.985	0.685
Team 51	0.717	0.828	0.769
Team 52	0.692	0.834	0.757
Team 57	0.703	0.875	0.780
Team 58	0.667	0.730	0.697
Our method	0.704	0.879	0.782

### Visualization of attention mechanisms

**Fig. 7:**
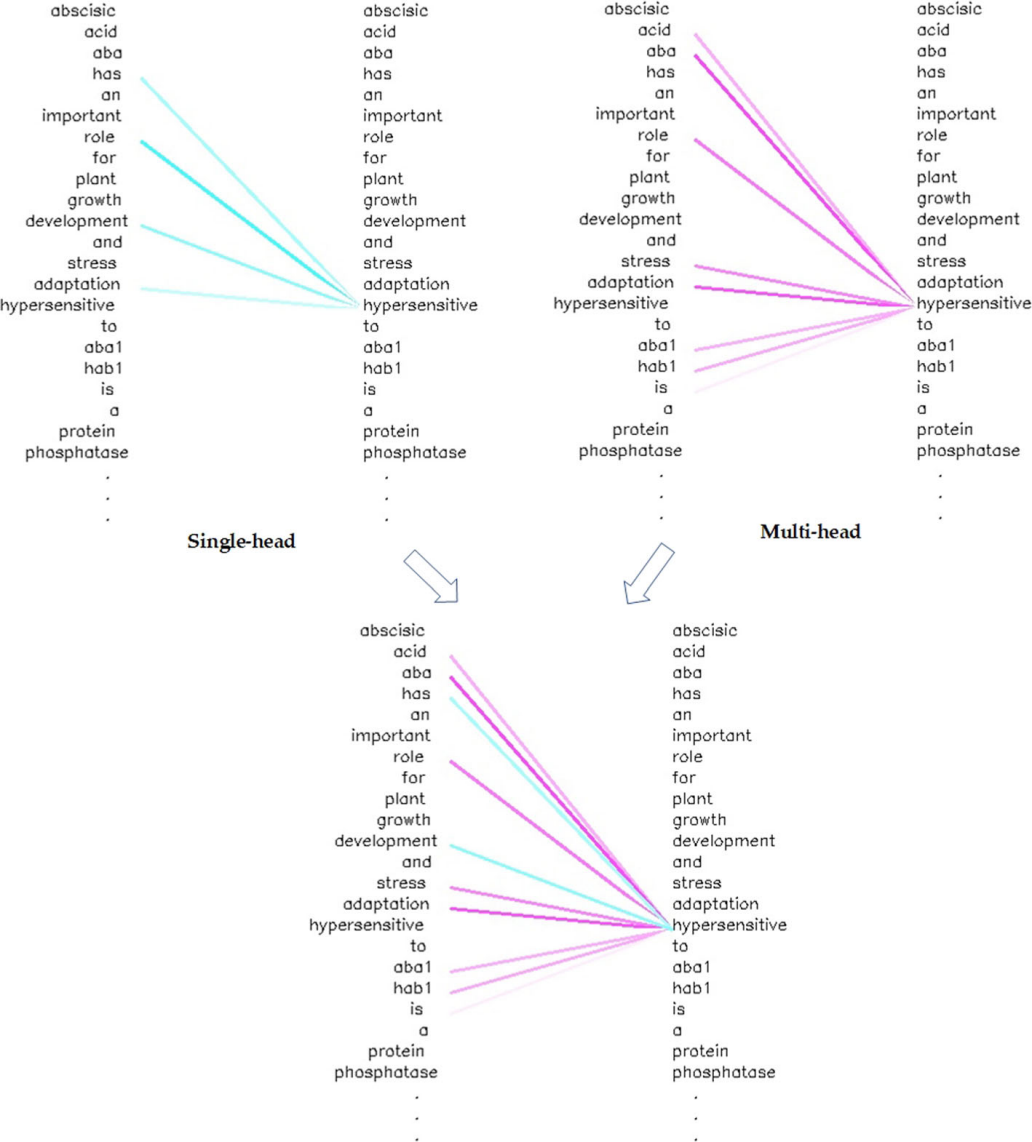
The visualization of the multi-head attention mechanism. The blue lines represent the dependence relationship between the words in the single-head self-attention mechanism and the purple lines represent the dependence relationship between the words in the multi-head self-attention mechanism

Considering that almost all the neural networks related to deep learning are used in the form of “black boxes”, we visualize the attention mechanism in order to increase the interpretability of the model. An example sequence is used to visualize the attention mechanisms in Fig. 7. The line connecting the two words represents the association of the two words. The deeper the color is, the closer the relationship between the two words will be. As can be seen from the figure, the self-attention mechanism can learn the dependence of the words in-side the sentence. The blue lines represent the dependence information of a single-head attention mechanism, from which we can find that the word ’hypersensitive’ can learn the dependence relationship with the word ’role’, ’has’, ’development’ and ’adaptation’. The purple lines represent the dependence information of another single-head attention mechanism, from which we can see that the word ’hypersensitive’ learns some different dependence relationship with different word ’acid’, ’aba’, ’role’, ’stress’, ’adaptation’, ’aba1’, ’hab1’, and ’a’. The multi-head attention mechanism in Fig. 7 combines the dependence information of the two single-head attention to extend the model’s ability to focus on different locations and provide multiple “representative subspaces” for the attention layer. It is possible to objectively observe the ability of self-attention mechanism finds the dependencies between words in a sentence by visualization of self-attention, indicating that there is an important position in the field of BioNLP for the attention mechanism. In addition, the attention mechanism is also one of the few deep learning models that can be visualized and can be analyzed in detail.

### Error analysis

We manually analyzed why our hierarchical attention-based capsule network model failed to better classify biomedical documents in the PM corpus experiments. The prediction results confusion matrix is shown in Table 8, from which we can see the number of false positives and true negatives for the classification error.

**Table 8:** The confusion matrix on PM corpus

Actual	Predict	
	True	False
True	865	142
False	522	98

**Fig. 8:**
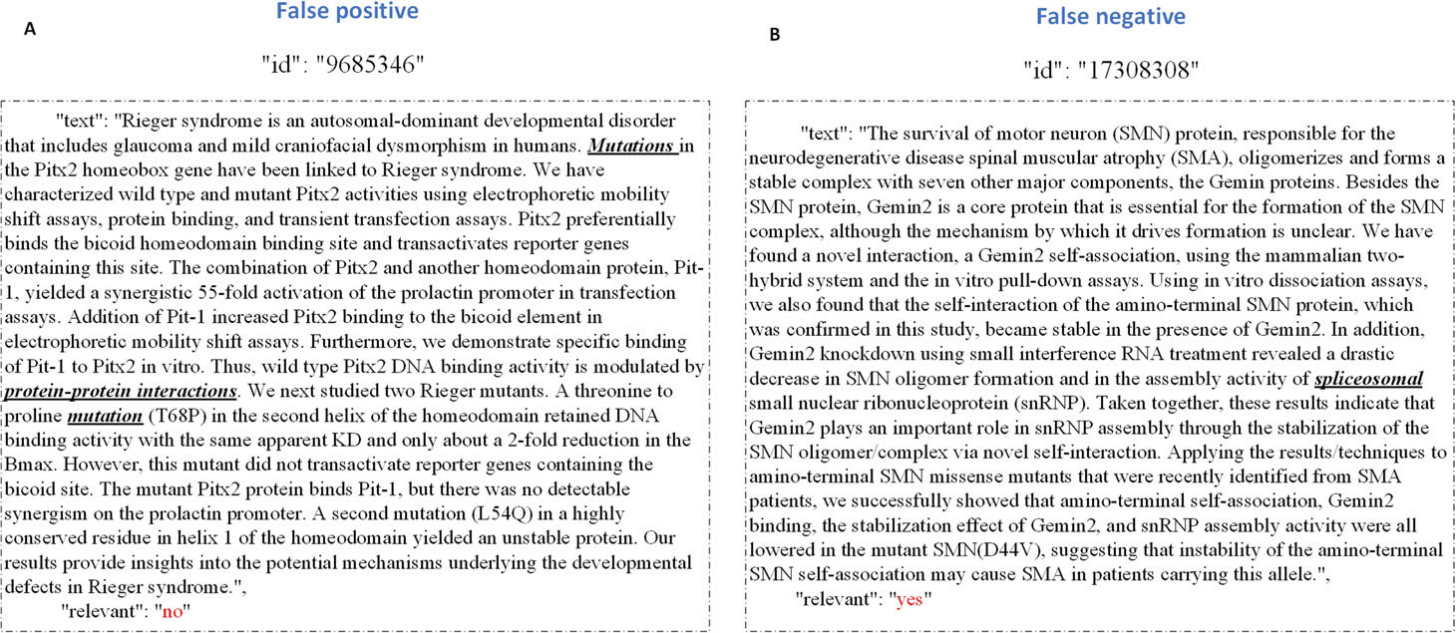
The false positive examples and false negative examples on PM corpus. **a** the false positive examples on PM corpus. **b** the false negative examples on PM corpus

We found that the significant classification error was the identification of negative examples as positive examples by our model. We analyzed the reason as follows. When an article included some strong PPI indicators or some words similar to the examples, our model mistook it as a positive example that is actually negative. Figure 8 gives the false positive examples, the words causing the misunderstanding are in bold. We can see that the text of PMID (PubMed ID): 9685346 contains some words such as protein, interaction, and mutation, which are strong positive keywords in PPIm articles, however, it does not describe PPI influenced by genetic mutations. Similarly, our model misclassified the PMID: 9685346 as a positive example because it has similar expressions with positive articles while it is actually negative.

When we analyzed the reason that the actual positives are deemed negatives, we found that some strong positive keywords were missing, or that the positive indicators did not appear in the positive articles. It is difficult to accurately classify true positive PPIm articles when their appearance is rare (even zero) in the training set. For example, the article with PMID: 17308308 actually describes PPI influenced by genetic mutations, but common positive keywords such as ’mutation’ are replaced by the words like ’spliceosomal,’ a word that rarely appears in the training set. Our model misclassified such positive instances as negative. In the future, a post-processing step could be helpful for these cases.

## Conclusion

Biomedical document triage is a crucial task in biomedical NLP, which is the first step in assisting literature curation workflows. Both attention mechanism and capsule networks are the recent advantages in neural networks. In this paper, we present a hierarchical attention-based capsule model for biomedical document triage. The proposed model employed the dynamic route algorithm and hierarchical attention mechanism to capture the important features across sentences. We evaluated our model on three BioCreative corpora. Experimental results showed that both hierarchical attention mechanism and capsule networks can improve performance in biomedical document triage. It is encouraging to see that our method achieved the state-of-the-art performance on all three corpora.

In future work, we will explore the effectiveness of pretrained deep contextualized word representations, such as Bert and ELMo, in biomedical document triage tasks. In addition, post-processing may further improve the performance of our model. The current state-of-the-art methods in biomedical document triage are primarily based on supervised machine learning and thus are highly dependent on sufficient labeled data. However, creating labeled datasets is prohibitively expensive and labor-intensive in the biomedical domain. Hence, reducing the dependency of methods on labeled training data is a key challenge in this domain. We will also plan on employing semi-supervised learning or transfer learning in biomedical document triage.

## Data Availability

PM, IAS and ACT data are all publicly available at https://biocreative.bioinformatics.udel.edu/resources/.

## Abbreviations

BioNLP: Biomedical Natural Language Processing
CNN: Convolutional Neural Networks
RNN: Recurrent Neural Network
PPIm: genetic mutations affecting Protein-Protein Interactions
PPI: Protein-Protein Interactions
PM: Precision Medicine
IAS: Interaction Article Sub-task
ACT: Article Classification Tasks
PMC: PubMed Central Open Access subset
LSTM: Long-Short Term Memory
